# ‘Pseudoneurological’ symptoms, dissociation and stress-related psychopathology in healthy young adults

**DOI:** 10.1186/1471-244X-13-149

**Published:** 2013-05-25

**Authors:** Petr Bob, Petra Selesova, Jiri Raboch, Lubomir Kukla

**Affiliations:** 1 Center for Neuropsychiatric Research of Traumatic Stress & Department of Psychiatry, First Faculty of Medicine of Charles University, Ke Karlovu 11, 128 00 Prague, Czech Republic; 2 Research Centre for Toxic Compounds in the Environment, Faculty of Science, Masaryk University, Brno, Czech Republic

**Keywords:** Alexithymia, Anxiety, Depression, Somatoform dissociation, Stress

## Abstract

**Background:**

Somatoform dissociation is a specific form of dissociation with somatic manifestations represented in the form of ‘pseudoneurological’ symptoms due to disturbances or alterations of normal integrated functions of consciousness, memory or identity mainly related to trauma and other psychological stressors. With respect to the distinction between psychological and somatoform manifestations of dissociation current data suggest a hypothesis to which extent mild manifestations of ‘pseudoneurological’ symptoms in healthy young population may be linked to stress-related psychopathological symptoms or whether these symptoms more likely could be attributed to unexplained somatic factors.

**Methods:**

With this aim we have assessed the relationship between somatoform dissociation and stress-related psychopathology (i.e. anxiety, depression, symptoms of traumatic stress, alexithymia) in a group of 250 healthy non-psychiatric and non-clinical young adults.

**Results:**

Results of this study show that the symptoms of somatoform dissociation are significantly linked to stress-related psychopathology.

**Conclusions:**

Findings of this study show that the ‘pseudoneurological’ symptoms may be linked to stress-related psychopathological processes which indicate that also mild levels of stress may influence somatic feelings and may lead to various somatoform dissociative symptoms.

## Background

Somatoform dissociation has been proposed as a concept describing specific forms of dissociative symptoms experienced as somatic disturbances due to alterations of normal integrative functions of consciousness, memory or identity related to stressful experiences [1-4]. Frequently these stressors are linked to an exposition of a trauma in childhood and related to physical, sexual or emotional abuse [5-8]. The somatic manifestations of dissociation are likely caused by a lack of integration of somatoform components of experience, reactions and functions and represented by various forms of pseudoneurological symptoms [8-11] involving bodily functions such as motor inhibition or loss of motor control, gastrointestinal symptoms, dissociative seizures, painful symptoms, alterations in perception or alterations in sensation of pain (analgesia, kinesthetic anesthesia), for example unability to register pain or painful affect during traumatic event [12-14]. Several studies have shown that the concept of somatoform dissociation may explain various somatic disturbances in psychiatric patients and also in patients with pain disorders that in many cases have unexplained etiology and in principle it could be related to stress exposure and related processes of mental disintegration [2,4,6,8-10,14]. As expected from the psychological theory and clinical data several findings also show that symptoms of somatoform dissociation have close relationship to psychologically experienced dissociative symptoms [6]. For example a recent study of young population of students strongly suggests that various stress factors related to dissociation may have direct and continuous relationship to somatic symptoms that may be explained within the concept of somatoform dissociation [15].

Although the concept of somatoform dissociation seems to be clinically relevant, the distinction between psychological and somatic forms of dissociation represents a fundamental problem whether dissociative symptoms, reflecting disorders of conscious awareness, are always “psychological” in nature or they may have somatic manifestations mediated by somatization or conversion mechanisms [6-8]. With respect to brain-mind reductionism that rejects mental causation the problem whether stress and traumatic experiences may cause only psychological or also somatic symptoms is still controversial [2,5-9,12]. This discussion in principle suggests clinically relevant empirical question and hypothesis whether mild manifestations of pseudoneurological symptoms linked to the concept of somatoform dissociation in general population may be attributed to stress-related psychopathological symptoms. Within this context, in somatically healthy people these symptoms likely cannot be explained by various underlying somatic factors.

With the aim to test the hypothesis we have assessed the relationship between ‘pseudoneurological’ symptoms represented by somatoform dissociation questionnaire, and stress-related psychopathological symptoms (i.e. anxiety, depression, symptoms of traumatic stress, alexithymia) in a group of 250 non-psychiatric and non-clinical healthy young adults, who represent population particularly vulnerable to stress influences.

## Methods

### Participants

Participants of this study were selected within the framework of European Longitudinal Study of Parenthood and Childhood (ELSPAC). The longitudinal study started in 1992 and included few European countries, and was organized in the UK and Czech Republic. Cohort of the study was selected randomly in the population of the city of Brno in the Czech Republic based on voluntary agreement provided by parents awaiting a newborn child. In the present study, as a part of ELSPAC, data of 250 non-psychiatric and non-clinical healthy young adults were collected. Based on anamnestic data exclusion criteria in this study were presence of psychiatric, neurological, internal and other somatic disorders. The participants were 101 men and 149 women (mean age 18.6 years old with high school education, age range within one year, more than 18 less than 19). Data characterizing participant’s somatoform dissociation and other psychopathological manifestations related to symptoms of traumatic stress, depression, anxiety and alexithymia were acquired. All the participants gave written informed consent and the study was approved by Masaryk university ethical committee. All the data used in the study were acquired at the Institute of Preventive and Social Pediatrics, Faculty of Medicine, Masaryk University, Brno and data acquisition and processing were done by mental health professionals at the Institute.

### Psychometric measures

Somatoform dissociative symptoms were assessed using the 20-item self-reported somatoform dissociation questionnaire SDQ-20 [9,16,17]. Somatoform dissociative symptoms represent alterations in sensations of pain (analgesia, kinesthetic anesthesia), alterations of perception, loss of motor control, gastrointestinal symptoms, etc. Subjects indicate the degree of their experience on 5-point Likert scale (total score from 20 to 100). In the study we have used the Czech version of the SDQ-20 that displays high reliability and internal consistency (Cronbach’s alpha 0.91, test-retest reliability after one week 0.90).

For investigation of childhood traumas, TSC-40 (Trauma Symptom Checklist) [16-18] was used. TSC-40 is a self-reported 40-item questionnaire done on a 4-point Likert scale (total score from 0 to 120). TSC-40 evaluates symptomatology in adult individuals associated with childhood or adult traumatic experiences and measures aspects of posttraumatic stress and other symptom clusters found in some traumatized individuals represented by subscales for dissociation, anxiety, depression, sexual abuse trauma index (SATI), sleep disturbances and sexual problems. The Czech version of the TSC-40 has high reliability and internal consistency (Cronbach’s alpha 0.91, test-retest reliability after one week 0.88).

For the assessment of depressive symptoms Czech version of Beck depression inventory BDI-II [16,17,19] was used that represents 21-items questionnaire for assessing depression (Cronbach’s alpha 0.89,test-retest reliability after one week 0.85). Subjects indicate degree of their experience of depressive symptoms on 4-point Likert scale (total score from 0 to 63).

Levels of anxiety symptoms were assessed using the Czech version of the Zung Self-Rating Anxiety Scale (SAS) (Cronbach’s alpha 0.89, test-retest reliability after week 0.85) [20,21]. The SAS is 20-item self-reporting questionnaire focused on the most common general anxiety symptoms. Each question is scored on 4-point Likert scale from 1 to 4 (total score from 20 to 80).

Alexithymia was assessed using the Czech version of the 20-item Toronto Alexithymia Scale (TAS-20) (Cronbach’s alpha 0.81, test-retest reliability after 1 week 0.77) [21,22]. Each question is scored on a five- point Likert scale from 1 to 5 (total score from 20 to 100).

The Czech versions of the all questionnaires were originally created in 2005 by translation from the English original and then back-translated into English and the resulting document was compared with the original by a native English speaker. The Czech version were then tested on a sample of 400 persons selected from general population and on samples of psychiatric and neurological patients, and results using these tests were published [16,17,21].

### Statistical methods

Statistical evaluation for the results of SDQ-20 and other psychometric measures included descriptive statistics, Mann-Whitney test for independent samples, Kruskal-Wallis ANOVA, Spearman correlation coefficients and multiple linear regression analysis. The non-parametric analyses were preferred because SDQ-20 data have not normal distribution. All the methods of statistical evaluation were performed using the software package Statistica version 6. To prevent Type II error which would disable to reject null hypothesis that symptoms of somatoform dissociation are not linked to stress-related psychopathological symptoms we performed Power Analysis and assessed the effect sizes characterizing differences between means of the subsamples with higher and lower levels of somatoform dissociative symptoms.

## Results

To test psychopathological symptoms with respect to occurrence of pseudoneurological symptoms related to somatoform dissociation we have used descriptive statistics for the whole sample (N = 250; Table 1) and then the participants were divided into two groups according to values of SDQ-20, i.e. higher (N1 = 130; SDQ-20 ≥ 22) and lower (N2 = 120; SDQ-20 < 22) than median. Based on this separation the results of the Mann-Whitney test show that the participants with higher level of symptoms of somatoform dissociation (SDQ-20) than median display increased level of symptoms of traumatic stress (TSC-40), depression BDI-II, anxiety (SAS) and alexithymia (TAS-20) in comparison to participants who have lower SDQ-20 score than median (Table 2). In the power analysis we have tested significant differences between means which show that with exception of TAS-20 with medium effect size (r from 0.3 to 0.5) all differences between means had strong effect size (r = 0.5 or higher; Table 2).

**Table 1:** Descriptive statistics for the whole group of participants (N = 250)

	**Mean**	**Median**	**Min.**	**Max.**	**Interquartile range**	**S.D.**
**SDQ-20**	23.22	22.00	20.00	51.00	5.00	4.35
**TSC-40**	17.70	15.50	0.00	56.22	16.00	11.31
**BDI-II**	7.71	5.00	0.00	33.00	9.90	6.95
**SAS**	33.27	32.00	20.00	69.00	10.00	8.05
**TAS-20**	45.98	45.00	23.00	74.00	12.00	9.35

*Note*. Somatoform Dissociation Questionnaire (SDQ-20); TSC-40- Trauma Symptoms Checklist; BDI-II- Beck Depression Inventory; Zung’s Self-Rating Anxiety Scale (SAS); Toronto Alexithymia Scale (TAS-20).

**Table 2:** Between group comparison for the participants with higher and lower SDQ-20 (than median)

	**Mean lower**	**Mean higher**	**MW- test Z**	**MW- test p**	**r**
	**<22 N = 130**	**≥22 N = 120**			
**SDQ-20**	20.44 ± 0.66	26.21 ± 4.66	−13.42	0	1.11
**TSC-40**	12.47 ± 8.36	23.36 ± 11.38	−7.79	10^−14^	0.96
**BDI-II**	5.62 ± 6.3	9.97 ± 6.94	−5.40	0.00000006	0.58
**SAS**	30.09 ± 5.81	36.70 ± 8.73	−6.40	0.0000000001	0.77
**TAS-20**	43.81 ± 8.09	48.32 ± 10.07	−3.51	0.0004	0.47

*Note*. Mean lower (or higher) represents mean score in the subgroup with SDQ-20 score lower or higher (or equal) with respect to median of SDQ-20; Somatoform Dissociation Questionnaire (SDQ-20); TSC-40- Trauma Symptoms Checklist; BDI-II- Beck Depression Inventory; Zung’s Self-Rating Anxiety Scale (SAS); Toronto Alexithymia Scale (TAS-20); r- standardized effect size- type I error rate alpha < 0.05.

The results also indicate that SDQ-20 is significantly correlated to TSC-40 (Spearman r = 0.545, p < 0.01), BDI-II (Spearman r = 0.419, p < 0.01), SAS (Spearman r = 0.471, p < 0.01) and TAS-20 (Spearman r = 0.307, p < 0.01). These correlations show that SDQ-20 pseudoneurological symptoms exhibit significant and proportional relationship to symptoms of traumatic stress, depression, anxiety and alexithymia. In the analysis we have not found specific relationships between SDQ-20 and the subscales of the TSC-40 and TAS-20. All correlations between SDQ-20 and the subscales were statistically significant but did not show statistically significant differences between the correlation coefficients.

To confirm and further analyze results obtained by correlation analysis we have divided the whole sample according to SDQ-20 score with respect to interquartile range (Table 1) into 4 subgroups 1^st ^from 20 to 25; 2^nd ^from 25 to 30; 3^rd ^from 30 to 35; and 4^th ^including all SDQ-20 scores from 36. To analyze differences in TSC-40, BDI-II, TAS-20 and SAS between the subgroups defined according to criteria of SDQ-20 subgroups we have used Kruskal-Wallis ANOVA. The results show that increased SDQ-20 scores in these subgroups are significantly linked to increased values of psychopathological symptoms measured by TSC-40, BDI-II, TAS-20 and SAS (z > 5.9; p < 0.00000001; H > 21.8) which indicate that increased values of somatoform symptoms are related to continuously increased psychopathological symptoms.

To analyze effects of TSC-40, BDI-II, TAS-20 and SAS on SDQ-20, we have used a multiple linear regression that may be useful to know whether stress-related psychopathological symptoms in their specific interactions are proportionally linked to increased levels of SDQ-20. The result shows that multiple R = 0.60 is statistically significant (p < 0.01; F = 34.44) which enables to define SDQ-20 as a linear function of four variables SDQ-20 = *f* (TSC-40, BDI-II, TAS-20, SAS).

## Discussion

Results of this study show that the ‘pseudoneurological’ symptoms described in the context of somatoform dissociation are significantly and proportionally associated with stress-related psychopathology and that also relatively mild stressors may be linked to somatic manifestations. A limitation of this study may be occurrence of “medically unexplained symptoms” [23] that may manifest in general population and in principle these symptoms may also have other explanations than somatic form of dissociation and statistically may influence the results. Other significant limitation with respect to the results and their interpretation is that correlation does not mean causality and in fact the processes related to stress and specific changes in the nervous system have characteristic quality of circular causality which means that brain vulnerability or dysfunction most likely creates higher sensitivity to stress influences and on the other hand through conversion mechanism psychological stress may influence the brain and its sensitivity with respect to various insults and other environmental influences.

Nevertheless with respect to the hypothesis findings of this study show that the “pseudoneurological” symptoms likely at least in part have psychological origin and can be explained by dissociative mechanisms in their somatic form within the framework of somatoform dissociation. Although some somatic factors influencing the symptoms cannot be rejected, predominant influence on these pseudoneurological symptoms likely may be linked to stress. Important aspect of this association presents the relationship between the pseudoneurological symptoms and alexithymia suggesting that loss of inner ability to distinguish, experience and interpret internal emotional states and feelings typical for alexithymia [19] is linked to the process of disordered conscious awareness related to the process of dissociation in its somatic form [6,24].

In addition, dissociated mental states have sensory, emotional and cognitive elements that may be misinterpreted and experienced as physical, and due to this misinterpretation physical and emotional experience can become confused and one of them may turn into the other [8]. For example, localized pain may depend on the reactivation of a previously dissociated traumatic memory linked to sensorimotor responses during the past traumatic experience which caused a lack of integration of somatoform experiences, reactions and functions [6].

This influence of stress and dissociation on somatic experience and bodily functions is in agreement with growing evidence that for example orbital prefrontal areas regulate affect, motivation, and bodily state and that early relational traumatic stress is specifically imprinted into the right brain, which is dominant for autobiographical memories [25]. Those and other scientific findings show that the mind–brain mechanisms present complex network, in which the brain is linked to the dynamics and entity called ‘mind’ that mediates subjective mental experience [26,27].

## Conclusion

Results of this study indicate that also mild levels of stress in the mind of healthy people may be linked to somatic pseudoneurological symptoms and that somatoform dissociation may present an important mediating factor which may explain relationship between mental stress and somatic symptoms. Results of the present study together with other reported findings strongly suggest that clear diagnostic distinction based on available evidence about somatoform dissociation presents an important issue for clinical practice. Mainly these research findings could be useful for description and classification of diseases that in their current forms (ICD-10, DSM IV) only partially take into account influences of mental states on somatic functions and symptoms. A detailed analysis which somatic symptoms might be with high probability attributed to somatoform dissociation likely would have high clinical impact for differential diagnostics and could provide useful diagnostic instrument.

## Competing interests

The authors declare that they have no competing interests.

## Authors’ contributions

PB, LK, JR- study design and data analysis; PS- collecting data; PB- wrote the paper. All authors read and approved the final manuscript.

## Pre-publication history

The pre-publication history for this paper can be accessed here:

http://www.biomedcentral.com/1471-244X/13/149/prepub
